# The Natural History of Biocatalytic Mechanisms

**DOI:** 10.1371/journal.pcbi.1003642

**Published:** 2014-05-29

**Authors:** Neetika Nath, John B. O. Mitchell, Gustavo Caetano-Anollés

**Affiliations:** 1 Biomedical Sciences Research Complex and EaStCHEM School of Chemistry, University of St. Andrews, North Haugh, St. Andrews, Scotland, United Kingdom; 2 Evolutionary Bioinformatics Laboratory, Department of Crop Sciences, University of Illinois, Urbana, Illinois, United States of America; Wellcome Trust Sanger Institute, United Kingdom

## Abstract

Phylogenomic analysis of the occurrence and abundance of protein domains in proteomes has recently showed that the α/β architecture is probably the oldest fold design. This holds important implications for the origins of biochemistry. Here we explore structure-function relationships addressing the use of chemical mechanisms by ancestral enzymes. We test the hypothesis that the oldest folds used the most mechanisms. We start by tracing biocatalytic mechanisms operating in metabolic enzymes along a phylogenetic timeline of the first appearance of homologous superfamilies of protein domain structures from CATH. A total of 335 enzyme reactions were retrieved from MACiE and were mapped over fold age. We define a mechanistic step type as one of the 51 mechanistic annotations given in MACiE, and each step of each of the 335 mechanisms was described using one or more of these annotations. We find that the first two folds, the P-loop containing nucleotide triphosphate hydrolase and the NAD(P)-binding Rossmann-like homologous superfamilies, were α/β architectures responsible for introducing 35% (18/51) of the known mechanistic step types. We find that these two oldest structures in the phylogenomic analysis of protein domains introduced many mechanistic step types that were later combinatorially spread in catalytic history. The most common mechanistic step types included fundamental building blocks of enzyme chemistry: “Proton transfer,” “Bimolecular nucleophilic addition,” “Bimolecular nucleophilic substitution,” and “Unimolecular elimination by the conjugate base.” They were associated with the most ancestral fold structure typical of P-loop containing nucleotide triphosphate hydrolases. Over half of the mechanistic step types were introduced in the evolutionary timeline before the appearance of structures specific to diversified organisms, during a period of architectural diversification. The other half unfolded gradually after organismal diversification and during a period that spanned ∼2 billion years of evolutionary history.

## Author Summary

Structural phylogenomics enables one to construct a historical timeline of the structural scaffolds known as protein folds and of the biocatalytic mechanisms that are embedded in them. This timeline defines a natural history of biocatalysis through its most granular components, the mechanistic steps. This history reveals an explosive diversity of catalytic mechanisms, which are used in a combinatorial manner in the different chemical reactions of the emergent metabolic networks. This evolutionary “big bang” of mechanistic innovation of protein reaction chemistries was based on mechanistic steps that were probably recruited from primordial chemistries that already existed on Earth, contributing uniquely and very early to life's nascent metabolic repertoire. This can benefit our understanding of protein structure–function relationships and of the origin of modern biochemistry.

## Introduction

The three-dimensional (3D) atomic structures of contemporary proteins provide clues about how both structure and function unfolded in the course of billions of years of evolution [1]. The phylogenomic analysis of protein domain occurrence and abundance in modern proteomes [2], [3] enables retrodictive views of protein evolution that are unanticipated [4], [5] and can be used to study structural change and the relationship between protein structure and function [6]. Two recent studies of this kind showed congruently that the α/β architecture is probably the oldest type of fold design [2], [3].

An interesting observation [3], [7], regarding the Enzyme Commission (EC) [8] definition of the overall function of enzymes, is that the oldest fold structures were associated with the largest number of enzyme functions [3], [7], [9], [10]. The EC classification provides functional annotations that can be used to link a gene with the chemical reaction catalysed by its gene product. However, the EC classification does not explore the detailed chemical mechanism of the enzyme reaction. Indeed, the classification was designed before much information concerning enzyme structures [11] and mechanisms [12], [13] was available.

Understanding how enzymes adapt their chemical mechanisms under evolutionary pressure is still a challenging task in molecular biology. In this study, we explore the chemical mechanisms used in biochemical reactions catalysed by ancestral enzymes. We ask questions about the ways in which enzyme structure and chemical mechanism have evolved together, and about the evolutionary origination of new enzyme structures and new catalytic mechanisms. MACiE [12], [13] definitions of enzyme mechanisms and ages of domain structures (MANET) [14] derived from phylogenomic analyses of protein structure [3], [5], [15] dissected the evolutionary appearance of novel structures and functions. It has been suggested that the difficulty of evolving novel stepwise chemical reaction mechanisms could be the dominant factor limiting the divergent evolution of new catalytic functions in related enzymes [16]. We put this concept to the test with phylogenomic analysis of protein domain structure and careful annotations of reaction mechanisms. Our observations have important implications for the origins of modern biochemistry and for exploring structure-function relationships.

## Methods

### Phylogenomic analyses

Biocatalytic mechanisms operating in metabolic enzymes were traced along an evolutionary timeline of appearance of domain structures defined at the homologous superfamily (H) level of structural abstraction of CATH [11]. Hereafter, we refer to these fold superfamilies as H-level structures. CATH unifies domain structures hierarchically from bottom to top into sequence families (SF), homologous superfamilies (H), topologies (T), architectures (A) and classes (C). H-level structures are considered evolutionary units. The timeline was built directly from a phylogenomic tree describing the evolution of 2,221 H-level structures [5], treating their phylogeny as monophyletic. The tree was reconstructed from a census of domains in 492 fully sequenced genomes (42 archaea, 360 bacteria and 90 eukarya). The census produced a data matrix of multistate characters coded alphanumerically with columns representing proteomes (phylogenetic characters) and rows representing H-level structures (phylogenetic taxa), which was used to build rooted phylogenomic trees in PAUP* version 4.0b10 [17]. Trees were reconstructed using the maximum parsimony (MP) method with 1,000 replicates of random taxon addition, tree bisection reconnection (TBR) branch swapping, and *maxtrees* unrestricted. Character states in the data matrix were polarized from ‘*N*’ to ‘*0*’ using the ANCSTATES command of PAUP*, where ‘*N*’ indicates the plesiomorphic (ancestral) state. The model of phylogenetic character transformation that was used assumes that domain age is in general proportional to domain abundance in proteomes. The biological basis for global increases in domain abundance is the existence of processes of gene duplication, amplification and rearrangement in genomes [18] that drive molecular innovation. Details and support for character argumentation have been presented previously [3], [15]. Since genomic abundance should be considered a natural evolving ‘heritable’ trait, trees are expected to be unbalanced. Indeed, trees of domain structures are highly unbalanced and follow a molecular clock of folds that links molecular evolution with the geological record [4]. Consequently, the relative age of a domain fold structure (*nd* value) was calculated directly from trees using a PERL script that counts the number of nodes from the ancestral structure at the root of the tree to each leaf and provides it on a relative zero-to-one scale. Using the molecular clock converts this relative evolutionary timeline into a truly temporal geological timeline expressed in billions of years. An *nd* value of 0 indicates the origin of proteins approximately 3.8 billion years ago and the oldest domain, and a value of 1 the present and the youngest domain structure.

Our phylogenetic methodology relates to definitions of structures that are modern, based upon a structural census in the proteomes of extant organisms. Consequently, retrodictions are derived from modern structural complexity and do not necessarily depict the actual structure of hypothetical ancestors, which will always remain unknown (molecules can be brought back from the past experimentally by resurrection but cannot be confirmed to be truly bona fide retrodictive constructs). However, if molecules become structurally canalized in evolution, then modern retrodictive statements truly approximate molecular history.

### Definition of molecular mechanism

**Figure 1:**
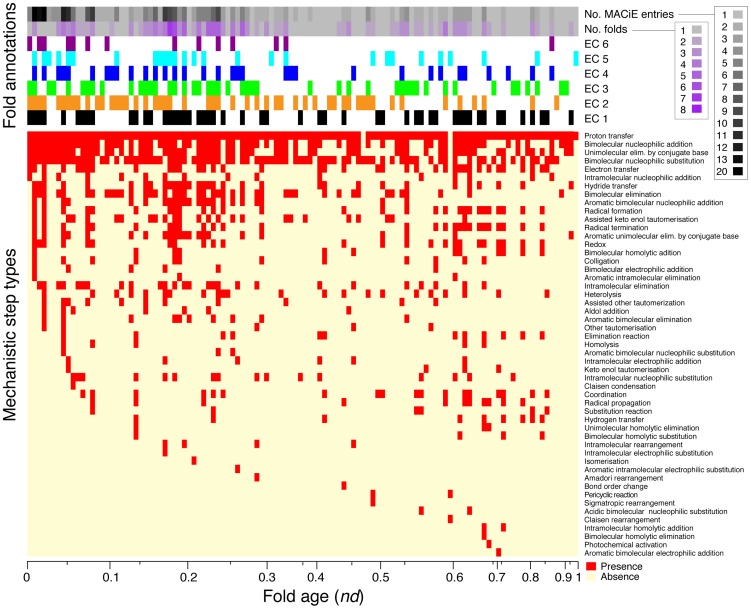
The history of biocatalytic mechanisms. The heat map describes the distribution of presence (red) and absence (yellow) of mechanism step types (y-axis) over fold age (x-axis). Rows of the heat map (mechanisms) are ordered vertically according to the first appearance of the step type in time, with the oldest at the top. The row sidebars at the top of the heat map are used to describe the number of MACiE entries and CATH H-level domain structures (annotated as number of folds) appearing at each fold age, and presence of top-level EC classes that are associated with these H-level structures (see color key). The x-axis scale reflects the different *nd* values found in our dataset, arranged from the oldest on the left to the youngest on the right. Every unique *nd* value forms a separate column. The non-linear scale is defined by the number of unique *nd* values falling in each interval of *nd*. There are many distinct *nd* values between 0.0 and 0.3 found in our dataset, so the scale is expanded in this region. There are few distinct *nd* values between 0.7 and 1.0, so the scale is very condensed in that region. Geological time is taken to be approximately linear with *nd*, where *nd* = 0 represents the origin of the protein world approximately 3.8 billion years ago and *nd* = 1 corresponds to the present [4].

**Figure 2:**
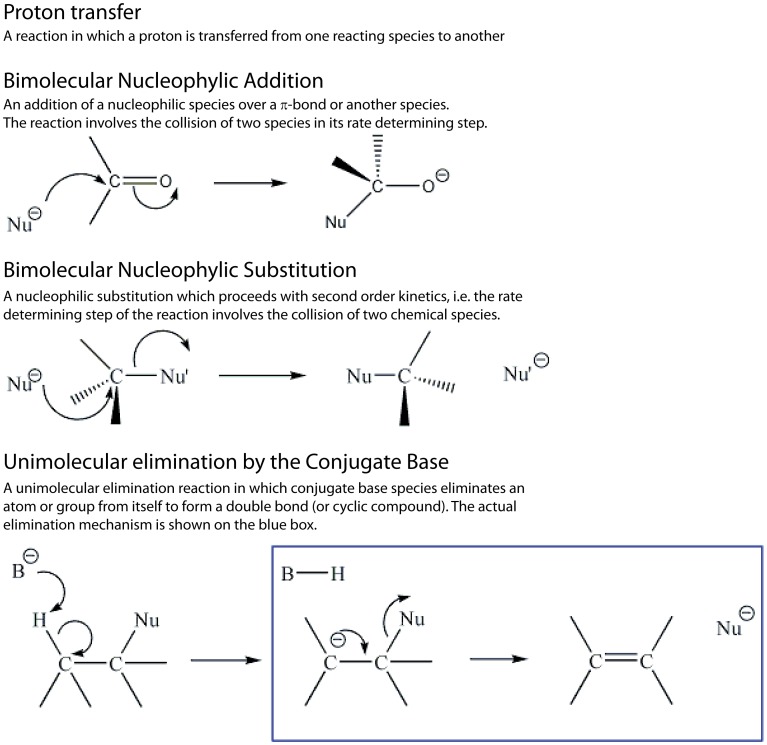
Definition of the most ancient mechanistic step types, which include fundamental building blocks of enzyme chemistry: “Proton transfer”, “Bimolecular nucleophilic addition”, “Bimolecular nucleophilic substitution”, and “Unimolecular elimination by the conjugate base”. We follow MACiE's terminology, though the latter could perhaps be better described as “Unimolecular elimination *from* the conjugate base”, being the second and last step of the E1cB “Unimolecular elimination *via* the conjugate base” mechanism.

For enzyme function definitions we have retrieved data from the MACiE database, specifically the functional annotations describing the chemical nature of individual reaction steps; frequently observed examples are “Proton transfer” and “Bimolecular nucleophilic substitution” (adundances and definitions in **Figures 1** and **2**, respectively). These MACiE annotations relate specifically to the steps of the mechanisms by which the reactions occur, rather than to the overall chemical transformation; the EC number covers the latter. To test the hypothesis of the ancestral folds using the most mechanistic step types, we retrieved 335 enzyme reactions from MACiE [19] version 3.0, mapped over fold age [5] using data from MANET [14]. MACiE is designed to be as complete as possible at the 1^st^, 2^nd^ and 3^rd^ levels of EC, but only representative at the 4^th^ level. Its coverage, relative to the numbers of nodes for which PDB structures exist, is 6/6 (1^st^ level); 54/57 (2^nd^ level); 165/194 (3^rd^ level); 249/1547 (4^th^ level), according to figures collated in 2010 [19]–[21]. In this study, we are using detailed mechanistic stepwise information extracted from the primary literature by the curators of MACiE.

### Data culling

Out of 335 MACiE enzyme reaction entries, 321 entries had unique overall functions at the 4th level of the EC classification. MACiE entries included catalytic domains which adopted 236 different structures, as indicated by CATH H-level structures, and received age assignments. We emphasise that we are specifically considering domains annotated in MACiE as catalytic. In many enzymes, not all domains were actually involved in catalysis. For example, MACiE enzyme reaction M0124 (EC 1.9.3.1, cytochrome c oxidase) was annotated with 16 domains, of which only one domain (CATH 1.20.210.10, cytochrome c oxidase chain A) was annotated in MACiE as a catalytic domain used to effect the reaction. So we included only one of the 16 CATH domains in this analysis, CATH 1.20.210.10. The catalytic domain distribution of the remaining enzyme structures was as follows: 240 enzyme entries with a single catalytic domain, 63 enzymes having two different catalytic domains, four enzymes with three catalytic domains and only one enzyme entry in MACiE (M0207, EC 2.7.9.1, pyruvate-phosphate dikinase) with four domains (CATH 3.30.1490.20, *nd* = 0.0539; CATH 3.30.470.20, *nd* = 0.058; CATH 3.20.20.60, *nd* = 0.112; CATH 3.50.30.10, *nd* = 0.377) that participate in catalysis; pyruvate-phosphate dikinase is a key enzyme participating in gluconeogenesis and photosynthesis. Thus, a total of 308 MACiE enzymes were considered for further analysis. Only these H-level structures were used further to explore the evolution of biocatalytic mechanisms.

### Annotation of domain structure and mechanism

Once the data were filtered, we associated H-level structures with the mechanistic step types, MACiE's annotations of the reaction steps catalysed by the corresponding enzymes. In this study, we used 51 mechanism annotation definitions from the MACiE database, which can be associated with the steps defined for the enzyme-catalyzed reactions. The data matrix was a presence and absence (PA) matrix where each column represents the occurrence of a “mechanistic annotation” and each row represents a fold with its corresponding fold age. For example, M0017 purine-nucleoside phosphorylase (CATH 3.40.50.1580, *nd* = 0.235) has only one domain and uses four reaction steps to complete its reaction. In order to effect the reaction, this enzyme goes through: step 1, “Proton transfer”; step 2, “Heterolysis”; step 3, “Bimolecular nucleophilic addition”; and lastly step 4, “Proton transfer”. In this analysis, “Proton transfer” was counted once for this enzyme. The glossary of the mechanistic step types can be found on the MACiE website (http://www.ebi.ac.uk/thornton-srv/databases/MACiE/glossary.html).

In cases where the enzyme had only one catalytic domain, we associated the mechanistic annotations of each step with the structure of the domain. In cases where enzymes used more than one domain to effect the reaction, we carefully selected the domain or domains participating in each step and issued the mechanistic annotation to the corresponding H-level structures. We assigned the mechanistic annotation only if at least one residue from the domain was catalytically involved in the corresponding reaction step in MACiE, either as a “Reactant” or as a “Spectator” [22]. The complete data culling process was done using an R script [23] for retrieving data from the MACiE database that filtered and mapped the 308 MACiE enzymes onto their relative fold ages.

## Results and Discussion

### A general approach grounded in protein domain structure

In order to test the hypothesis that the most ancestral protein domains use the greatest number of biocatalytic mechanistic step types, we assume that extant protein domain structure is the best historical archive that is available to explore ancient enzyme functions. The assumption holds good ground. At high levels of structural complexity, evolutionary change occurs at an extraordinarily slow pace. A new fold superfamily may take hundreds of thousands to millions of years to materialize in sequence space while new sequences develop on Earth in less than microseconds [24]. In fact, a recent comparative analysis of aligned structures and sequences showed that structures were 3–10 times more conserved than sequences [25]. Here we use the ages of domain structures, derived from phylogenomic reconstruction and a recent census of CATH domain structure in hundreds of genomes [5], to study how chemical mechanisms developed in protein evolution. The use of molecular structure and abundance in phylogenomic analysis offers numerous advantages over traditional methods [26], eliminating phylogenetic problems such as alignment, phylogenetic inapplicables and taxon sampling. Their use does not violate character independence, a serious problem that has not been addressed in phylogenetic sequence analysis. To our knowledge, this is the first study to explore the evolution of biocatlytic mechanisms using a timeline of CATH homologous superfamily (H-level) domain structures and data analysis. However, there is another comprehensive database, FunTree [27], that brings together sequence, structure from CATH, chemical and mechanistic information from MACiE, and phylogenetics.

### Historical trends unfold a natural history of biocatalytic mechanisms

In order to explore the use and reuse of biocatalytic mechanisms in evolution, we mapped the mechanistic definitions of enzymatic functions to their respective CATH H-level structures, with structures ordered according to fold age (**Figures 1** **,** **3** **,** **4**). For this purpose we first created a presence and absence (PA) matrix, a heat map representing the distribution of the presence (red) and absence (yellow) of the mechanistic step types (rows, y-axis) in the fold (columns, x-axis) (**Figure 1**). The rows were ordered vertically according to the first appearance of the mechanism over fold age and were indexed with the numbers of: (i) MACiE enzyme entries (shades of grey and black), (ii) H-level structures (shades of grey and purple), and (iii) EC classes that appeared at each age. The complete data set is provided as Supporting Information, **Dataset S1**.

**Figure 3:**
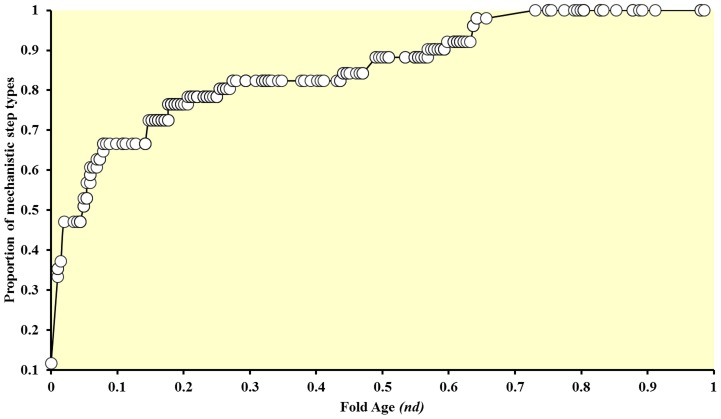
Cumulative plot describing the appearance of mechanistic step types in protein domain evolution. The graph shows the proportion of mechanistic step types that are present at a particular time.

**Figure 4:**
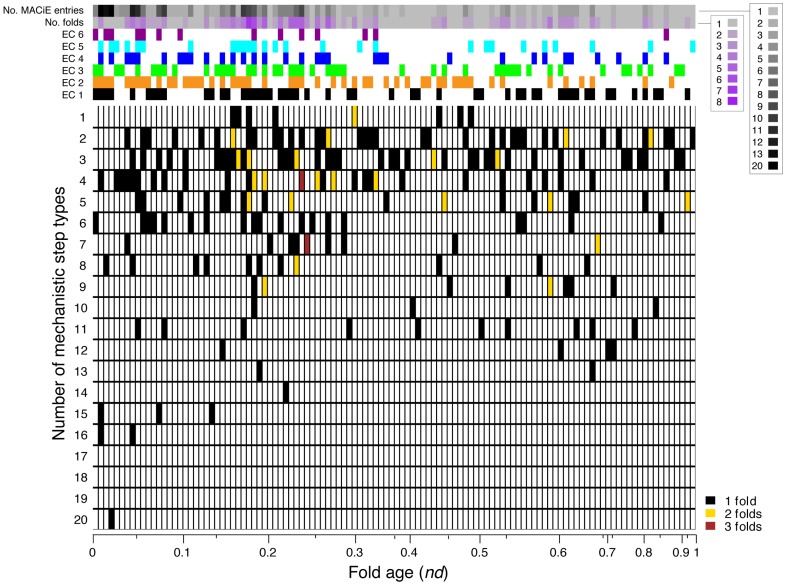
Heat map representing the number of mechanistic step types (y-axis) used by H-level structures of each different fold age (x-axis). Different colors indicate distinct structures which happen to share both the same number of mechanistic step types and an identical fold age. For example, in column 2 the black coloring of rows 4, 15 and 16 shows that four structures respectively accommodate 4, 15 and 16 different mechanistic step types to effect their reactions. The color code for the row sidebar is similar to that in Figure 1; the x-axis scale is also similar to that in Figure 1.

Remarkably, the most popular enzyme mechanistic step types were associated with the oldest H-level structures (**Figure 1**). This evolutionary trend suggests that the oldest enzymes already provided a sufficiently flexible scaffold to support many diverse mechanistic step types in order to complete their reactions. Within the early scaffolds, the mechanistic steps had more time to be adapted by the domain structures and to be further recruited in the course of evolution. The existence of late emerging structures with many mechanistic steps supports the presence of widespread recruitment processes in evolution. This trend seems to be explained in terms of the “*preferential attachment principle*” that guides the growth of scale-free network behavior, and implies that the more prevalent functions are typically the earliest, as previously shown in the exploratory analysis of the ancestral fold structures [28].

We observed that “Proton transfer”, “Bimolecular nucleophilic addition”, “Bimolecular nucleophilic substitution”, and “Unimolecular elimination by (or from) the conjugate base” (definitions are represented in **Figure 2**) are the most common mechanistic step types, in accordance with their distribution in MACiE enzyme reaction mechanisms (the prevalence of each step type is also given in Supporting Information, **Table S1**) [12], [29]. These types of mechanistic steps are recognisably fundamental building blocks of enzyme chemistry, which is carried out in aqueous solution usually at approximately neutral pH. Several of the canonical amino acids have pKa values close to neutral, with Holliday *et al*. having observed particularly strong propensities for His and Glu to facilitate proton transfer [12]. The chemistry of the amino acid side chains also means that several are negatively charged at roughly neutral pH, and hence it is no surprise that the enzyme far more often acts as a nucleophile, favoring mechanisms labelled as nucleophilic, rather than as an electrophile. Furthermore, it has been noted that enzyme active sites are well suited to stabilising the charged intermediates common in addition and elimination reactions, for instance by hydrogen bonding [22]. The ubiquity of aqueous environments in enzyme chemistry restricts the repertoire of reactions available. Indeed, most enzyme reactions are composed of steps that might seem unexciting to an organic chemist. The rare occurrence of more complicated organic chemistry, “Aldol addition”, “Amadori rearrangement”, “Claisen condensation”, “Claisen rearrangement”, “Pericyclic reaction” and “Sigmatropic rearrangement”, constitutes the exception rather than the rule, and enzymes sample the space of possible mechanisms notably differently from how an organic chemistry textbook would do so.

The rate of introducing new mechanistic step types at different fold ages is shown in **Figure 3**, which represents a cumulative plot where fold age is shown on the x-axis. The y-axis shows the proportion of the total number of defined step type annotations (*N* = 51) that have been uncovered up to that fold age on the x-axis. It is clear in this plot that the first four H-level structures (the first two increments of fold age, 0 to 0.0098 ) are responsible for introducing a third of the known mechanistic step types (18/51), and the first six structures (the first four increments of fold age, 0 to 0.049) are responsible for over half of them (27/51). However, the development of the other half was harder and required the unfolding of about ¾ of the evolutionary timeline, up to *nd* = 0.73, and about 2.5 billion years of evolution (inferred using a molecular clock of folds [4]). The detailed information regarding the introduction of mechanistic step types is provided in **Table 1** **.**

**Table 1:** Discovery of MACiE's mechanistic step types according to the evolutionary timeline of domain structure innovation.

Fold age	CATH	Description	Mechanisms discovered
**0**	3.40.50.300	P-loop containing nucleotide triphosphate hydrolases	Bimolecular nucleophilic addition
			Bimolecular nucleophilic substitution
			Intramolecular nucleophilic addition
			Proton transfer
			Unimolecular elimination by the conjugate base
			Electron transfer
**0.0098**	3.40.50.150	Vaccinia Virus protein VP39	Bimolecular elimination
**0.0098**	3.40.50.720	NAD(P)-binding Rossmann-like Domain	Bimolecular elimination
			Aromatic bimolecular nucleophilic addition
			Aromatic unimolecular elimination by the conjugate base
			Assisted keto-enol tautomerisation
			Aromatic intramolecular elimination
			Bimolecular homolytic addition
			Radical formation
			Radical termination
			Redox
			Bimolecular electrophilic addition
**0.0098**	3.50.50.60	FAD/NAD(P)-binding domain	Bimolecular elimination
			Aromatic bimolecular nucleophilic addition
			Aromatic unimolecular elimination by the conjugate base
			Assisted keto-enol tautomerisation
			Aromatic intramolecular elimination
			Bimolecular homolytic addition
			Radical formation
			Radical termination
			Colligation
			Redox
**0.0147**	3.40.50.620	HUPs	Intramolecular elimination
**0.0196**	3.20.20.70	Aldolase class I	Heterolysis
			Aldol addition
			Assisted other tautomerisation
			Aromatic bimolecular elimination
			Other tautomerisation
**0.0490**	3.40.50.970	Not Assigned (1-deoxy-D-xylulose-5-phosphate synthase -like domain 1/2/3)	Homolysis
			Elimination reaction
**0.0490**	3.40.190.10	Periplasmic binding protein-like II	Aromatic bimolecular nucleophilic substitution
**0.0539**	3.90.226.10	2-enoyl-CoA Hydratase; Chain A domain 1	Keto-Enol tautomerisation
			Intramolecular electrophilic addition
**0.0588**	3.40.47.10	Peroxisomal Thiolase; Chain A, domain 1	Claisen condensation
**0.0588**	3.40.30.10	Glutaredoxin	Intramolecular nucleophilic substitution
**0.0686**	3.60.21.10	Purple Acid Phosphatase; chain A, domain 2	Coordination
**0.0784**	2.60.120.10	Jelly Rolls	Radical propagation
**0.0784**	3.40.50.1820	Not Assigned 4,9-DSHA hydrolase activity, (Carboxyesterase-related protein -like domain 1)	Substitution reaction
**0.1471**	3.20.70.20	Anaerobic Ribonucleotide-triphosphate Reductase Large Chain	Bimolecular homolytic substitution
			Hydrogen transfer
			Unimolecular homolytic elimination
**0.1765**	1.10.600.10	Farnesyl Diphosphate Synthase	Intramolecular electrophilic substitution
			Intramolecular rearrangement
**0.2059**	2.40.100.10	Cyclophilin	Isomerisation
**0.2549**	3.40.50.10090	Not Assigned (Uroporphyrinogen-III synthase -like domain 1/2)	Aromatic intramolecular electrophilic substitution
**0.2745**	3.30.1130.10	GTP Cyclohydrolase I, domain 2	Amadori rearrangement
**0.4412**	1.10.520.10	Not Assigned (Catalase-peroxidase -like domain 1/2)	Bond order change
**0.4902**	3.40.50.10230	Precorrin-8X methylmutase CbiC/CobH	Sigmatropic rearrangement
			Pericyclic reaction
**0.5686**	1.10.606.10	Vanadium-containing Chloroperoxidase domain 2	Acidic bimolecular nucleophilic substitution
**0.5980**	1.10.590.10	Chorismate Mutase subunit A	Claisen rearrangement
**0.6373**	3.20.20.240	TIM Barrel	Intramolecular homolytic addition
			Bimolecular homolytic elimination
**0.6422**	1.25.40.80	Serine Threonine Protein Phosphatase 5, Tetratricopeptide repeat	Photochemical activation
**0.7304**	1.10.800.10	Phenylalanine Hydroxylase	Aromatic bimolecular electrophilic addition

First column represents nd values, second CATH code, third CATH H-level structure names (in cases where the names were not assigned, we have given the FunFams description) and the last column represents mechanistic step types as described in MACiE.

In order to look at the distribution of the mechanistic step types of an enzyme in evolutionary time, we counted the number of mechanistic step types associated with H-level structures (**Figure 4**). **Figure 4** is a heat map representing the number of mechanism step types (y-axis) used by those structures having each different discrete value of fold age (x-axis). Each cell represents the number of H-level structures with a different color code; for example black represents 1 structure, yellow represents 2 structures and brown represents 3 structures sharing the same count of mechanistic step types. Moreover, each position indicates the number of H-level structures associated with a number of functions. For instance, black color at column 1 row 6 means that there is one structure that uses 6 different mechanistic step types to complete its reaction. The x-axis scale reflects the different *nd* values found in our dataset, arranged from the oldest on the left to the youngest on the right. Every unique *nd* value forms a separate column. The non-linear scale is defined by the number of unique *nd* values falling in each interval of *nd*. In a further section, we will discuss the patterns in detail.

### Ancient H-level structures are popular, central and versatile

The most ancient H-level structure that appears in the MACiE database is CATH 3.40.50.300, the P-loop containing nucleotide triphosphate hydrolase. This fold has been consistently identified as the most ancestral fold structure [2], [3], [5]. The P-loop hydrolase structure consists of the most ancient and abundant topology, the Rossmann fold (CATH 3.40.50), which has the 3-layer (αβα) sandwich (3.40) architecture. The CATH 3.40.50.300 superfamily contains enzymes with diverse molecular functions, including signal transduction, hydrolase and transferase enzymatic activities [30]. Wang *et al*. previously observed [15] diverse overall functions for this structure (the complete list of MACiE enzyme entries is given in Supporting **Dataset S1**). In the current analysis, there are only five MACiE enzyme entries that share this structure; these are associated with six mechanistic step types, “Proton transfer”, “Electron transfer”, “Bimolecular nucleophilic addition”, “Bimolecular nucleophilic substitution”, “Intramolecular nucleophilic addition” and “Unimolecular elimination by the conjugate base” (**Table 1**). MACiE enzymes associated with this oldest structure are dethiobiotin synthase (EC 6.3.3.3, M0074), estrone sulfotransferase (EC 2.8.2.4, M0154), H^+^-transporting two-sector ATPase (EC 3.6.3.14, M0178), nitrogenase (EC 1.18.6.1, M0212, multi-domain) and adenylate kinase (EC 2.7.4.3, M0290). Except for nitrogenase, the rest of these enzyme entries each have a single catalytic domain, hence, it is straightforward to annotate the function with this fold. Nitrogenase (M0212, PDB: 1n2c) [31] is a very important enzyme of nitrogen metabolism that fixes atmospheric nitrogen (N_2_) gas into the reduced forms that are usually assimilated by plants [32]. The enzyme has a complex 3D structure that is highly conserved across many different organisms and contains domains from three different homologous superfamilies. These H-level structures first evolved at different times. The ancient CATH 3.40.50.300 nitrogenase catalytic core was later accesorized with a domain from the CATH 3.40.50.1980 superfamily, which evolved at *nd* = 0.401 after the oxygenation of Earth's atmosphere [4], [33], [34], and a non-catalytic domain CATH 1.20.89.10, which appears to have been accreted last into the molecule (*nd* = 0.549). Residues from the ancient nitrogenase core with the oldest domain of the molecule are involved in the first two steps of the long 15-step reaction, which include the mechanistic step types “Bimolecular nucleophilic substitution”, “Electron transfer” and “Proton transfer”. The remaining 13 steps are carried out by catalytic residues from the CATH 3.40.50.1980 domain.

The three H-level structures at the second most ancient fold age include CATH 3.50.50.60, the T-level topology of which is 3-layer ββα; its H-level structure has no specific name assigned, but corresponds to the FAD/NAD(P)-binding domain FunFams definition in CATH and is found in 7 MACiE entries. Having the same fold age, we find CATH 3.40.50.720 (NAD(P)-binding Rossmann-like domain) in 12 MACiE enzymes, and CATH 3.40.50.150 (Vaccinia Virus protein VP39) in two MACiE entries. All three H-level structures appear at *nd* = 0.0098. These structures have 16, 15, and 4 catalytic mechanistic step types (**Figure 4**), respectively, of which a total of 11 are non-overlapping with those of the first P-loop hydrolase fold structure and were therefore newly introduced at this time (see **Table 1**). These newly evolved mechanistic step types include three involving aromatic groups, as well as the first involving radicals, and also “Bimolecular electrophilic addition”, “Bimolecular elimination”, “Redox”, “Colligation” and “Assisted keto-enol tautomerisation”. It was interesting to note that the “Bimolecular elimination” mechanism was shared by all three H-level structures of the same age. There are 9 different mechanisms shared by CATH 3.40.50.720 and CATH 3.50.50.60 (shown in **Table 1**). Studies by the Orengo group [35], [36] suggest there may be distant homology between these structures, based on their similarity in graph-based structure comparison and shared use of organic cofactors (NAD and FAD). The structures are functionally diverse due to the conformational change of the ligands, organic cofactors or structural plasticity of the proteins [37].

In MACiE, the ferredoxin-NADP^+^ reductase enzyme (M0142, EC: 1.18.1.2) combines the CATH 3.40.50.150 and CATH 3.50.50.720 H-level structures to complete its biochemical reaction. This enzyme plays a very important role in electron transfer from the flavoenzyme NADPH-adrenodoxin-reductase (AdR) to two P450 cytochromes; this process is involved in the production of steroid hormones. The two domains of this enzyme share the following functions: “Aromatic unimolecular elimination by the conjugate base”, “Aromatic bimolecular nucleophilic addition”, “Redox”, “Radical termination”, and “Radical formation”.

The next most ancient H-level structure (*nd* = 0.0147), CATH 3.40.50.620, the H-level Hups α/β layered fold, is responsible for 13 MACiE entries and introduces the novel “Intramolecular elimination” function. This structure supports central catalytic functions of the cell, including the aminoacylation reactions of aminoacyl-tRNA synthetase (aaRSs) catalytic domains that are crucially involved in the attachment of L-amino acids to cognate tRNA molecules and are responsible for the specificity of the genetic code. The structure includes the tyrosyl-tRNA ligase EC function (M0197; EC 6.1.1.1) of the tyrosyl-RS functional family, the oldest aaRSs delimiting the process of translation [38]. The enzyme activates a specific amino acid by condensation with ATP to form an aminoacyladenylate intermediate, which then esterifies the 2′ or 3′-hydroxyl group of the ribose at the 3′ end of the acceptor arm of tRNA. The aminoacylation site rejects larger amino acids and a proofreading site in an editing domain hydrolyzes small amino acids that were incorrectly activated through pre-transfer or post-transfer editing mechanisms.

### Some structures hold exceptionally diverse mechanistic step types

Some H-level structures by nature use many diverse mechanistic step types to effect their catalytic activity. A member of the TIM barrel α/β structure that is highly popular in metabolism, the CATH 3.20.20.70 superfamily (aldolase class I, *nd* = 0.0196), which immediately follows the aaRS fold in the timeline, supports a diversity of chemistry that includes 20 different mechanistic step types. Five of these appeared for the first time with this fold (**Table 1**). It is not surprising that the fold has such diverse functions. Based on the Hierarchic Classification of Enzyme Catalytic Mechanisms (RLCP; where R: Basic Reaction, L: Ligand group involved in catalysis,C: Catalysis type and R: Residues/cofactors located on Proteins) classification [39] analysis of functional subclasses [40], Nagao *et al*. suggested that aldolase class I enzymes have various functional classifications. An interesting conserved property is that most of their ligands have at least one phosphate group. The mechanistic step types of aldolase class I (see **Table 1**) are rare in the MACiE database. Out of 335 MACiE enzyme entries, “Aldol addition”, “Aromatic bimolecular elimination”, “Assisted other tautomerisation”, “Heterolysis” and “Other tautomerisation”, respectively, appeared in 9, 6, 20, 25 and 9 MACiE enzyme entries in at least one stage of the reaction (the numbers of different MACiE entries containing each of the mechanistic step types are given in **Table S1**). This suggests that the aldolase class I superfamily contains a group of enzymes that possess very specific mechanistic step types.

Two additional H-level structures utilise 16 different mechanistic step types each, CATH 3.50.50.60 (*nd* = 0.0098) (which we have already mentioned) and CATH 3.40.50.970 (*nd* = 0.049), the second largest number of mechanistic step types associated with any structures in the timeline. These structures also belong to the most popular fold topology, the Rossmann fold. Following their appearance (*nd* = 0.049), most of the fundamental and common mechanistic step types had already been introduced. The CATH 3.40.50.970 structure introduces “Homolysis”, represented in only one MACiE entry (M0119; EC: 1.2.7.1; pyruvate: ferredoxin oxidoreducatse). We observed that two mechanistic step types, “Homolysis” and “Colligation”, were introduced at the same fold age but by different H-level structures. By definition, the “Homolysis” mechanistic annotation is the converse of the “Colligation” step that was introduced by CATH 3.50.50.60; “Homolysis” is the cleavage of a covalent bond where each atom retains one of the two bonding electrons, whereas “Colligation” is when two free radicals combine to form a covalent bond.

### The combinatorics of mechanistic steps reveals winners

**Figure 5:**
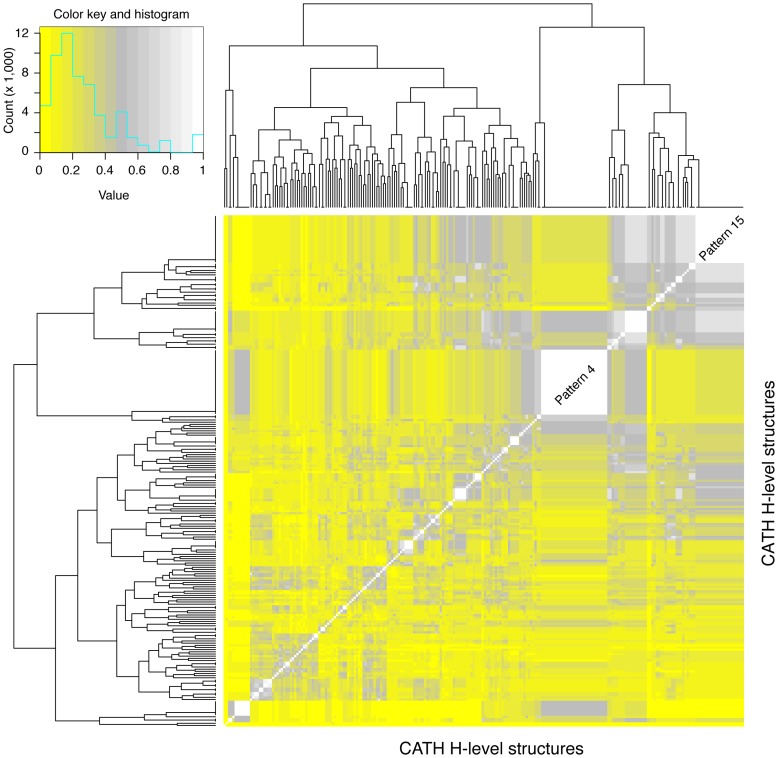
Heat map representing the similarity of mechanistic step types utilised by the H-level structures. For this we have calculated the Jaccard similarity scores. Here the x and y axes in the plot are ordered using a hierarchical clustering algorithm in which the two most similar data points are linked together at each iteration. The colors of the heatmap represent the similarity scores where yellow suggests low or no (when 0) similarity and white (1) means that identical combinations of mechanistic steps are shared between two H-level structures. The top left corner represents the color key for the similarity scores and the distribution of the similarity scores.

We were also interested to see what sets of mechanistic step types described the combinations of steps used by various enzymes to effect their reactions. To do so, we looked for the combination of the different mechanistic step types, irrespective of order, and at the various H-level structures sharing each combination of biochemical steps. Instances of reutilisation of particular mechanistic step types may shed light on evolutionary recruitment of common mechanistic steps by different structures. For this we first created “mechanistic annotation patterns”. These patterns reflect all the different combinations of the presence and absence of mechanistic step types. This kind of analysis illustrates that different H-level structures share common mechanistic annotation patterns. We found that there are 133 different mechanistic annotation patterns used by the enzymes in our dataset (the complete mechanistic annotation patterns are provided in the Supporting Information, **Table S2** and **Table S3**). Pattern 4 is most popular mechanism combination, involving “Bimolecular nucleophilic substitution” and “Proton transfer” (see **Figure 5**, H-level structures are grouped together in the white box). There are 42 H-level structures in MACiE that use two mechanistic step types in order to complete their reactions. Out of these 42 structures, 30 use pattern 4 in order to complete their reactions. Patterns 4 and 15 suggest that there are few H-level structures (details of superfamilies and pattern association are represented in Table S3) that accommodate similar mechanistic step type combinations.

Pattern 15 is the second most popular pattern and includes “Bimolecular nucleophilic addition”, “Proton transfer” and “Unimolecular elimination by the conjugate base”. In MACiE, there are 46 different catalytic H-level structures that use three mechanistic step types in order to complete their reactions, out of which 22 structures use pattern 15 to effect their reactions. The enzymes of the CATH 3.20.20.70 (aldolase class I) structure use the maximum number of 20 different mechanistic step types to effect their overall reactions. These step types constitute pattern 133 (see **Table 2**), which is not shared by any other structure. These patterns suggest which mechanistic step types are compatible with one another or are preferentially combined together. There are 101 patterns unique to one structure (see **Table S3**).

**Table 2:** Pattern 133, the mechanistic step types associated with CATH 3.20.20.70, Aldolase class I.

Mechanistic step types with CATH 3.20.20.70, Aldolase class I
Unimolecular elimination by the conjugate base
Redox
Radical termination
Radical formation
Proton transfer
Other tautomerisation
Intramolecular nucleophilic addition
Intramolecular elimination
Hydride transfer
Heterolysis
Electron transfer
Bimolecular nucleophilic substitution
Bimolecular nucleophilic addition
Bimolecular elimination
Assisted other tautomerisation
Assisted keto-enol tautomerisation
Aromatic unimolecular elimination by the conjugate base
Aromatic bimolecular nucleophilic addition
Aromatic bimolecular elimination
Aldol addition

To visualise the combinatorial patterns, we have plotted a heat map of similarity of the mechanistic step types between two H-level structures (**Figure 5**). We calculated the Jaccard similarity scores; 
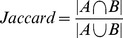
where *A* and *B* are two sets and the Jaccard coefficient of similarity is defined as the size of the intersection divided by the size of the union between the two sets. To visualize computed similarity scores, we constructed a presence and absence (PA) matrix where columns represent the mechanistic annotation as an entity and rows represent the CATH H-level structures. The score ranged from 0 to 1, with 0 signifying that no similar mechanistic step types existed between two structures and 1 signifying that the two structures shared an identical combination of mechanistic step types in order to complete their reactions. The most popular mechanism combinations, pattern 4 (“Bimolecular nucleophilic substitution” and “Proton transfer”) and pattern 15 (“Bimolecular nucleophilic addition”, “Proton transfer” and “Unimolecular elimination by the conjugate base”), are labelled in the heat map of **Figure 5** and are clearly distinguishable. As expected, these patterns include the most common and ancient mechanistic step types introduced with the CATH 3.40.50.300 structure.

The research goals of this paper are not to explore mappings of mechanistic step types along metabolic pathways, as this would require one to unfold a complex network structure with graph theoretical approaches. However, in order to make explicit the complex recruitment patterns that are expected we have mapped H-level structures in the nucleotide interconversion pathway of purine metabolism [41], the oldest of all metabolic subnetworks defined by the KEGG database [42]. Since nucleotide interconversion precedes purine biosynthesis in evolution [41], we compared mechanistic step types associated with this pathway (**Table 3**). In MACiE, we found only 8 H-level structures involved in purine metabolism, ranging in *nd* value from 0 to 0.411. Remarkably, and despite the absence of MACiE entries for the most ancient enzymes of energy interconversion (EC 2.6.1.3. and EC 3.6.4.1), the results reveal the very early rise of the highly abundant pattern 4 in evolution and complex patterns of recruitment of additional chemistries (**Figure S1**) which are ultimately associated with the combinatorics of mechanistic step types of **Figure 5**.

**Table 3:** MACiE enzymes for purine metabolism.

MACiE	Enzyme Name	EC	Subnetwork	PDB	CATH H level Structure	*nd value*	Combinatorial patterns	Mechanistic step types
**M0290**	adenylate kinase	2.7.4.3	INT	1zio	3.40.50.300	0	Pattern 2	Bimolecular nucleophilic substitution
**M0234**	GMP synthase (glutamine- hydrolysing)	6.3.5.2	INT	1gpm	3.40.50.880	0.0980	Pattern 4 (+2)	Proton transfer
								Bimolecular nucleophilic substitution
								Unimolecular elimination by the conjugate base
								Bimolecular nucleophilic addition
**M0326**	pyruvate kinase	2.7.1.40	INT	1pkn	3.20.20.60	0.1127	Pattern 4	Proton transfer
								Bimolecular nucleophilic substitution
**M0326**	pyruvate kinase	2.7.1.40	INT	1pkn	2.40.33.10	0.4118	Pattern 4	Proton transfer
								Bimolecular nucleophilic substitution
**M0080**	adenylosuccinate lyase	4.3.2.2	INT	1c3c	1.20.200.10	0.1667	Pattern 6	Proton transfer
								Bimolecular elimination
**M0065**	adenylosuccinate synthase	6.3.4.4	INT	1gim	3.40.440.10	0.2353	Pattern 4 (+2)	Proton transfer
								Bimolecular nucleophilic substitution
								Assisted other tautomerisation
								Aromatic bimolecular nucleophilic substitution
**M0150**	nucleoside-diphosphate kinase	2.7.4.6	INT	1ndp	3.30.70.141	0.3186	Pattern 4	Proton transfer
								Bimolecular nucleophilic substitution

Table columns are: MACiE code, Enzyme name, EC number, Purine metabolic subnetwork [41], PDB code, CATH H-level Structure, nd value and mechanistic step types.

### Conclusions

Contemporary protein structures consist of independently folding and compact domains that can be used as a fossil record of molecular evolution. We have utilised the available resources of enzyme mechanisms and the relative ages of CATH H-level domain structures to get a better insight into the natural history of biocatalytic mechanisms. Our analysis shows that the most designable structures (e.g., the α/β barrel and Rossmann fold) served as scaffolds to higher numbers of biochemical functions. The first two structures were responsible for introducing 35% (18/51) of the known mechanistic step types. Over half of these appeared in the evolutionary timeline of domains before structures specific to Archaea, Bacteria and/or Eukarya [5], during a period of architectural diversification (*nd*<0.39). The most common mechanistic step types were also the most ancient and included fundamental building blocks of enzyme chemistry, “Proton transfer”, “Bimolecular nucleophilic addition”, “Bimolecular nucleophilic substitution”, and “Unimolecular elimination by the conjugate base”. Later on in evolution, these mechanistic steps participated in a combinatorial interplay and were the highest represented in catalytic functions. The combination of “Bimolecular nucleophilic substitution” and “Proton transfer” was the most popular of all patterns of mechanistic step types. The other half of mechanistic step types appeared gradually after organismal diversification (0.67<*nd*<1) and during a period that spanned ∼2 billion years of evolutionary history.

Our phylogenomic approach is based on a census of protein domain structure in the proteomes of cellular organisms and the crucial axiom of polarization that claims that structural abundance increases in the course of evolution. This ‘process’ model of molecular accumulation in proteomes is based on Weston's generality criterion of homology and additive phylogenetic change [43] that in our case describes the slow and nested accumulation of homologous domain structures in the branches (proteome lineages) of the tree of life. A careful phylogenetic reconstruction analysis reveals that while both gains and losses of domain structures are frequent events, gains always overshadow losses in evolution [44]. This supports the general proportionality of domain abundance and evolutionary time of phylogenetic argumentation and the principle of continuity, the most important pillar of Darwinian evolution.

In these studies we trust the CATH classification scheme of domain structure, assignments of known structures to sequences, and current understanding of metabolic networks and associated chemical reactions. We note that it is highly likely that there is an ‘underground’ metabolism of weak catalytic specificities that is not annotated and involves a multiplicity of substrates and perhaps mechanistic step types. Our analysis is unable to capture this aspect of enzymatic function at this time. Similarly, our analysis does not explore biases in the distribution of annotations of molecular functions among structures and structures among functions nor the distribution of mechanisms across enzymatic reactions. Instead, it reveals patterns of accumulation of mechanistic step types in evolution.

The historical patterns we reveal uncover an explosive diversity of catalytic mechanisms embedded in the explosive discovery of EC functions [6], which are used in the different chemical reactions of the emergent metabolic networks. The evolutionary driver of mechanistic innovation of protein reaction chemistries was probably recruitment of strategies used in primordial metabolic chemistries that already existed on early Earth and their internalization into the emerging polypeptide scaffold. Support for this contention comes from a careful mapping of structures, functions and prebiotic chemical reactions in purine metabolism, the most ancestral metabolic subnetwork of metabolism [6]. This mapping revealed a gradual replacement of abiotic chemistries and the existence of concerted enzymatic recruitments driving the early evolution of pathways of nucleotide interconversion and the late appearance of pathways of biosynthesis, catabolism and salvage [41].

## Supporting Information

Figure S1 Early evolution of mechanistic step types in the most ancient of all metabolic pathways. The diagram describes structural and functional innovation and recruitment of enzymes participating in the nucleotide interconversion (INT) pathway of the purine metabolism subnetwork of KEGG. The diagram shows that pattern 4 of possible mechanistic step type combinations is the most popular choice among the enzymes of this ancient pathway. Among the mechanistic step types in pattern 4, “Proton Transfer” is used by almost all the enzymes in the subnetwork (see Table 3). Annotated H-level structures associated with enzymatic activities are traced in the pathways with a color code according to their *nd* value, which is also given in table format together with CATH H-level code and mechanistic step type patterns. The most ancient enzymes exhibit a number of additional mechanistic step types that add to those of pattern 4. These additional mechanistic step types are listed in parentheses (+x, where x represents the number of additional types). For details of H-level structure and pattern association, see Table S3.
(TIF)

Table S1 The mechanistic step type definitions, and the numbers and proportions of MACiE mechanisms that include each step type. The counts are from the complete MACiE data set (335 reaction mechanisms).
(XLSX)

Table S2 Patterns of mechanistic step types present in at least in one entry in MACiE.
(XLSX)

Table S3 Association between the CATH H-level structures and patterns of mechanistic step types. Patterns shared by more than one structure have their pattern numbers highlighted in green; patterns that are unique to one structure are not highlighted.
(XLSX)

Dataset S1 The complete data set used in our analysis, where the first column represents the fold age (*nd* values), the second column is the H-level CATH code, and subsequent columns contain the CATH description, MACiE entry number, Enzyme Commission number, and enzyme name. The MACiE entry numbers highlighted in red are the enzymes possessing metal co-factors.
(XLSX)
