# Supplementary figures and images for: The Natural History of Biocatalytic Mechanisms
Source: PLoS Comput Biol. 2014 May 29;10(5):e1003642. doi: 10.1371/journal.pcbi.1003642 (PMC4038463; doi:10.1371/journal.pcbi.1003642)

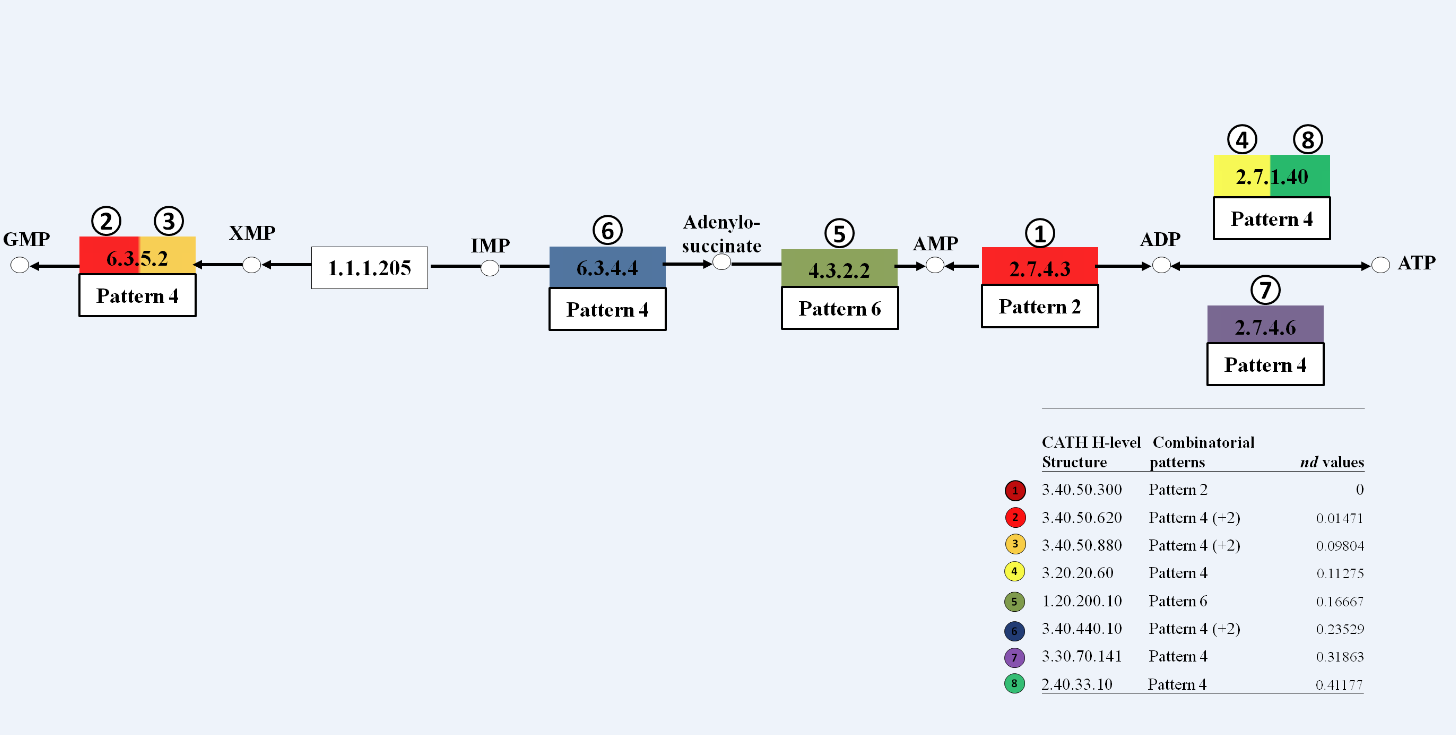

Supplement: Figure S1 — Early evolution of mechanistic step types in the most ancient of all metabolic pathways. The diagram describes structural and functional innovation and recruitment of enzymes participating in the nucleotide interconversion (INT) pathway of the purine metabolism subnetwork of KEGG. The diagram shows that pattern 4 of possible mechanistic step type combinations is the most popular choice among the enzymes of this ancient pathway. Among the mechanistic step types in pattern 4, “Proton Transfer” is used by almost all the enzymes in the subnetwork (see Table 3). Annotated H-level structures associated with enzymatic activities are traced in the pathways with a color code according to their nd value, which is also given in table format together with CATH H-level code and mechanistic step type patterns. The most ancient enzymes exhibit a number of additional mechanistic step types that add to those of pattern 4. These additional mechanistic step types are listed in parentheses (+x, where x represents the number of additional types). For details of H-level structure and pattern association, see Table S3. (TIF) [file pcbi.1003642.s001.tif]
